# A comprehensive home-care program for health promotion of mothers with preeclampsia: protocol for a mixed method study

**DOI:** 10.1186/s12978-019-0695-8

**Published:** 2019-03-14

**Authors:** Zahra Rastegari, Mohammad H. Yarmohammadian, Fatemeh Mohammadi, Shahnaz Kohan

**Affiliations:** 1 Student Research Center, Faculty of Nursing and Midwifery, Isfahan University of Medical Sciences, Isfahan, Iran; 2 Department of Health Management and Economic Research Center (HMERC), Medical Management and Information Sciences Faculty, Isfahan University of Medical Sciences, Isfahan, Iran; 3 Reproductive and sexual Health, Nursing and Midwifery Care Research Center, Faculty of Nursing and Midwifery, Isfahan University of Medical Sciences, Isfahan, Iran

**Keywords:** Home-care, Preeclampsia, Needs, Strategies, Mixed- methods study, Health promotion

## Abstract

**Background:**

One of the most common complications of pregnancy and the third cause of maternal death is preeclampsia. Thus this group of mothers should be supported, trained and received efficient health care services. Home-care is one strategy to improvement complications of Pregnancy. In Iran, high-risk pregnancies care provide in health care centers, hospitals and clinics by midwives and obstetricians. In this mixed method study, at first, a qualitative approach will be used to identify preeclampsia mothers’ health needs and home-care strategies for them. Then, the qualitative results will be emerged with literature review and expert ideas to develop a comprehensive home-care program which fits with the needs of these mothers in Iran.

**Methods:**

This study is a qualitative- quantitative mixed exploratory research that consists of three sequential phases. In the first step, in qualitative study, all the women with preeclampsia, obstetricians, midwives, and maternal health policy makers will select purposefully. Health care needs and home-care strategies for mothers with preeclampsia will be determined. Sampling continues until data saturation. In the second step, an expert panel will be formed to prioritization of home-care needs and strategies extracted from result of qualitative study and review of literature. Afterwards, Primary home care program will be designed. In the third step, Delphi method will be used of minimum 10–15 experts including: obstetricians, midwives, and reproductive health professionals about validation of this home-care program by questionnaires in three rounds, then the final program is being developed.

**Discussion:**

It is expected conducting a mixed method study to develop a home care program mothers with preeclamsia to improve their health status and wellbeing while reducing additional health care costs through preventing excessive admissions and interventions. Moreover it wants to follow up properly and timely high risk pregnant women. This study might be helpful in improvement quality of health services and promote health equity.

**Conclusion:**

Developing a home care program for maternal health care especially for high risk pregnancy by considering Iran socio-cultural context.

## Plain English summary

A home care pregnant woman is as a strategy for delivering services to improve health, wellbeing and education for mother with complication pregnancy. Because hypertensive disorders are second leading cause mortality and morbidity during pregnancy, home care for this vulnerable mothers has recommended to health promotion and improvement outcome pregnancy. This study is an exploratory mixed – method study (qualitative-quantitative) that will conduct in three sequential phases. The first phase with a qualitative approach, the researcher explores needs and strategies related to home care of women with preeclampsia. in the second phase, the researcher by combination prioritization result of expert panel and review of literature will design a primary home care program. In the third phase of the study, validating home care program will be investigated by Delphi method. After applying expert opinions, a comprehensive home care program for women with preeclampsia will be developed. a home care program mothers with preeclamsia to improve their health status and wellbeing while reducing additional health care costs through preventing excessive admissions and interventions. Moreover it wants to follow up properly and timely high risk pregnant women. This study might be helpful in improvement quality of health services and promote health .

## Background

Maternal health has always been one of the major health concerns of different communities. The reports of World Health Organization indicate that every day, approximately 830 women die from preventable causes related to pregnancy [8]. One of the main problems in maternal mortality and morbidity are high risk pregnancies [14]. Pregnancy-induced hypertensive disorders are considered to be the most common adverse outcomes of pregnancy all over the world. Annually, nearly 50,000 women worldwide die due to this health issue, And also similar to the same number experience other serious complications of these disorders, including stroke, renal failure and etc. [4]. It is also noteworthy that high blood pressure is an iatrogenic underlying cause for preterm birth [20].

In the past, preeclampsia was intervened with pregnancy termination, but current data indicate that prenatal outcomes improve with expected management. Moreover, the mean gestational age increase with expectation management and becomes closer to term pregnancy [22]. Although pregnant women with hypertensive disorder mostly experience hospitalization [9]. But many clinicians believe that further hospitalization is not warranted if hypertension abates within a few days. Therefore, Many women with mild to moderate hypertension can receive continuing care and manage at home [4].

In the early stages, regular monitoring along with precise and high quality interventions at home can reduce the complications of this related disorder and Providing an opportunity for counseling and training, and consequently improving pregnancy outcomes [17]. Furthermore, providing some parts of prenatal care at home reduce the stressors associated with these pregnancies and improve mental health in mothers with high risk pregnancy [5].

According to many benefits of home-care program, this strategy is being implemented in health delivery system in many developed countries to protect women with high-risk pregnancies.

Providing health care at home natural environment,allows the person to feel more control over her life status and simultaneously as well as receive a safe and supervised health care [6]. On the other hand,, with the training provided at home, pregnant women learn to change behaviors that decrease risk of preterm delivery and modify their health life style [16], also it is observed that assessment of mothers at home, minimize the number of days of hospitalization [1].

The provision of this care can lead to decrease premature birth and hospitalization costs in low birth weight neonate due to high risk pregnancy [11, 12]. Forcada reported that mothers who received home care besides prenatal routine care during pregnancy had less complication in pregnancy such as spontaneous premature rupture of membrane, preterm birth, and hypertension than patients treated only in the hospital [7].

Home - care program allow health care providers to take care of person in an environment where they feel comfortable and meet their family members. [23] moreover, maternal and fetal assessment, coordinating problem cases with health centers, educating the mother about high-risk situations and follow up their required service could achieve. [13] and so, this make easier for families to take part in educational programs and have a better attitude toward effective factors of maternal and child health [3]. By implementing a home- care program tangible psychosocial support, improvement medical communications and more using from social services will be conceivable [2].

In Iran, maternity care programs for pregnant mothers with hypertension focus more on treatment which are provided in health centers and specialized hospitals, It seems that continuity of these services are in some cases disorganize and pregnant women return to hospitals and health care centers with severe conditions [21].

Therefore, in order to reduce maternal mortality and complications in mothers with preeclampsia, the promotion of quality of care should be emphasized more strongly. Since home health care for pregnant mothers with preeclampsia is not currently available in the maternal health care system of Iran, this sequential mixed- study has been designed to develop an efficient home-care program based on the needs and social-cultural context of the country.

## Objectives

The objectives of each phase are as follows**:**

### Objectives of the first phase: Qualitative study:

1. identifying the home-care needs of mothers with preeclampsia
2. identifying strategies to access to home-care program for mothers with preeclampsia

### Objectives of the second phase: Quantitative study

1. Prioritizing home care needs in mothers with preeclampsia
2. Prioritizing the strategies to access home care program for mothers with preeclampsia
3. Design a primary home care program for mothers with preeclampsia

### Objectives of the third phase: Develop program

- Validate and Develop a comprehensive home care program for mothers with preeclampsia

## Methods/design

This study is a qualitative -quantitative mixed exploratory that consists of three sequential phases. In the first step, data collection will be carried out by qualitative approach, the researcher will explain needs and home care strategies to health promotion of women with preeclampsia. In this stage, all the women with preeclampsia, obstetricians, midwives, and maternal health policy makers are considered as the participants to understand and extract mother^’^s health care needs and the strategies for receiving home care services. In the second phase prioritization of needs and strategies of home-care program for mothers with preeclampsia will be performed, and then by combining the results extracted from qualitative data and review of literature, a primary home care program is designed. In the third phase, using Delphi approach, comments of at least 10–15 experts including obstetricians, midwives, and reproductive health professionals will be collected by questionnaires in three rounds for validating this program, and then according to expert opinions, a comprehensive home-care program will be developed for mothers with preeclampsia.

### First phase: Qualitative study

The first phase of this study is designed to answer the question “What are the needs and strategies for home care to promote health of women with preeclampsia?” This study will be carried out using a qualitative content analysis method.

### Participants in the first phase (qualitative study)

In the qualitative part of the present study, the research population will be considered to be the women with preeclampsia who referred to hospitals and health centers, obstetricians, reproductive health professionals, and maternal health policy makers, health providers who have experience in providing healthcare to women with preeclampsia that these people are selected with a purposeful sampling method and based on maximum variation (age, education, social status, job, economic conditions, and gestational age).

### Inclusion criteria for participants: a) women with preeclampsia will be included in the study by the following criteria

1. Having informed consent to participate in the research
2. being able to communicate and conduct interviews
3. Iranian citizenship and able to understand and speak in Persian
4. No history of well-known psychiatric disorder
5. The pregnant mothers with preeclampsia or a history of preeclampsia at previous pregnancy

### b) Health care providers and specialist entry into the study by the following criteria

1. Tendency to participate in the study with informed consent.
2. Having at least 12 months of experience in providing health care to women with preeclampsia history.

### Research environment

Selection of participants will be conducted in coordination and with the views of the participants at the time and place designated by them, wherever they feel comfortable (hospital, health centers, work places, university, home, etc.) or in places preferred by the participants.

### Data collection process in the first phase (qualitative study)

After obtaining permission from Isfahan University of Medical Sciences, the researchers will select the participants by referring to hospitals and health centers. The research makes necessary coordination and appropriate participants are selected with purposive sampling. After introducing, explaining the purpose of the study and the method of doing the research, the eligible participants are assigned an appointment in a private and comfortable environment. In the qualitative phase, data collection methods will be individual, in-depth, and semi-structured interviews along with taking notes in the fields. After The explaining objectives and methodology of the study, the researcher will receive written consent regarding participation in the research, for interviews, and recording them. If a person does not agree with recording, taking notes will be done. Interviews with pregnant mothers begin with some general questions: “tell us about your health problems during pregnancy”, and so “What kind of services do you receive?” and or “in your opinion, How you could receive these services at home?”

the questions which asked from obstetricians, health providers include: “What are your concerns when you send a mother with mild preeclampsia to home?” and so “How are health services delivered at the meantime for mothers with preeclampsia?” and or “From your point of view, how can these women receive these services at home?”, “and which kind of problems will the care givers be encountered and what are your suggestion?”.

And the questions which asked from reproductive health professionals and maternal health policy makers include: “What challenges do you encounter with preeclampsia mothers?” and also “According to your experiences, is it possible to design home-care programs for them?”

At the end of each interview, interviewer will listen to audio file carefully several times and afterward the narrative will be transcribed immediately, and data analysis will be done simultaneously with data collection. Data collection continues until the data saturation which means no new code or data is extracted.

### Data analysis of the first phase (qualitative study)

In the present study, the conventional content analysis Method will be used for data analysis [18]. After transcribing each interviews, the text will be read line by line, and its meaning units will be identified. Then, the sentences and the important phrases will be underlined, and the main ideas derived from them, labeled as codes.. After extracting the primary code, the data will be reduced, and eventually Subcategories, categories and the main categories respectively will appear from these codes.

### Rigor and trustworthiness of qualitative data

To ensure the accuracy of the study and the reliability of the results, four criteria are suggested: credibility, dependability, conformability and transferability. In order to increase the credibility of the study, participants will be selected with maximum variation, sufficient time will be assigned for data collection, and the integration of multiple data collection methods, such as interviewing and taking notes in the field will be used. Reviewing by the participants will be used to verify the accuracy of the extracted data and codes or to modify them.

And for confirmability of the findings, some examples of code extraction and their corresponding interview, narratives will be reviewed by an external supervisor in order to control the accuracy of researcher’s perception and to find contradictory cases. For increasing transferability, study findings will be presented to people who similar with participants in order to compare the results of this study with their own experiences. Also, the researcher will explain the whole procedure, including recording, transcription, code extraction, and categorization, In order to verify the coding procedure, some of the research colleagues and faculty members, who are acquainted with qualitative analysis are asked to review the procedure.

### Second phase: Quantitative phase

In the second phase of the study, after completing qualitative study, the researcher will start searching literatures and texts that address needs of mothers with preeclampsia and home care strategies. The review of literature in this research includes searching through library resources (reference books and theses) and searching electronic resources in order to access more information about the needs and home care strategies mothers with preeclampsia. At this stage, all studies with quantitative, qualitative or mixed method which have been published in the last 10 years in languages English and or Persian are examined with separate or combinational keywords, such as home-care programs, preeclampsia, and clinical guidelines. The researcher searches through databases such as web of science, Magiran, PubMed, science direct, Cochrane library, Scopus, Proquest, Ovid, SID, MEDLINE, Embase, and Google Scholar which have been published during 2008 to 2018 will be studied and analyzed. And afterwards qualitative study results and review of articles prepared to discuss at expert panel .

### Holding a panel of experts

At this stage, the intention is to prioritize the strategies extracted from qualitative study and review of literature. Obstetricians, reproductive health professionals, health services managers, as well as maternal health policy makers will form panel of experts (these people will be selected by the research team based on their professional experiences). In this step of the research, the decision matrix will be used to prioritize the needs and strategies extracted from literature review and qualitative study. In this matrix, a score between 1 and 3 will be designed for each strategy in terms of (cost, feasibility, time spent). Average rating is determined. After calculating The mean score of the strategies, the prioritized strategy is selected based on experiences and ideas of the team of experts. and a primary of home care program for mothers with preeclampsia will be prepared.

### Third phase: Developing and validation of comprehensive program

#### The study population

Target population for this stage is experts (obstetrician, reproductive health, providers,maternal health policy maker).

#### Research sample

In the third phase, this draft program will be sent for at least 10–15 of other experts in first Delphi rounds, the average score for each part will be determined and after the second and third Delphi round, the proposed program will be reviewed and evaluated at meeting with presence of the research team and panel members. The program will modify and reviewed in each stage based on the expert comments. Eventually, the modified and edited program will approved and the final comprehensive home care program will be developed.

## Discussion

Since the main purposes of the implementation and optimization of health care programs are prevention of high-risk pregnancies and reduce maternal mortality and morbidity, therefore designing interventions such as providing home care during pregnancy is important at this time. By implementation of this program, mothers can be continuously monitored and receive the necessary health care when faced with any specific problems at the earliest possible opportunity, and also This delivery health service can reduce the number of high-risk pregnancies, subsequent complications, and cesarean sections due to such pregnancies. Furthermore, it provide suitable opportunities for care givers to execute their training, interventional, evaluation, and supporting programs during pregnancy [19]. At the meantime, these programs are considered to be logical and efficient solutions for improving pregnancy outcomes [10]. Expansion of care to home is an appropriate strategy which improves mother-fetus health through increased awareness of mother and her family, social support, and providence of accessible health care [15]. Thus, this study aims to extract home-care needs of mothers with preeclampsia and present a comprehensive program that consistent with cultural and social necessities of the country. Since needs assessment is an indispensable component of programming, the needs are determined and prioritized, and consequently, the required data for programming will be extracted [24]. In sum, it is expected from the presented program of this study to make a clear path to design home-care programs of mothers with preeclampsia.

## Conclusion

Home care is a lost ring of perinatal care in Iran, which can play an important role in improving the health status and reducing the morbidity and mortality of mothers and infants, especially in high-risk pregnancies. And in order to ensure the design of services that are effective and consistent with the cultural context of the community and to prevent the loss of resources, it is essential to conduct numerous studies prior to any planning.
